# Assessing government policies' impact on the COVID-19 pandemic and elderly deaths in East Asia

**DOI:** 10.1017/S0950268822001388

**Published:** 2022-08-22

**Authors:** Cathy W. S. Chen, Mike K. P. So, Feng-Chi Liu

**Affiliations:** 1 Department of Statistics, Feng Chia University, Taiwan; 2 Department of Information Systems, Business Statistics and Operations Management, Hong Kong University of Science and Technology, Hong Kong

**Keywords:** Case fatality ratio, COVID-19, non-pharmaceutical interventions, elderly group, Spearman's rank correlation

## Abstract

This study assesses governments' long-term non-pharmaceutical interventions upon the coronavirus disease 2019 (COVID-19) pandemic in East Asia. It advances the literature towards a better understanding of when and which control measures are effective. We (1) provide time-varying case fatality ratios and focus on the elderly's mortality and case fatality ratios, (2) measure the correlations between daily new cases (daily new deaths) and each index based on multiple domestic pandemic waves and (3) examine the lead–lag relationship between daily new cases (daily new deaths) and each index via the cross-correlation functions on the pre-whitened series. Our results show that the interventions reduce COVID-19 infections for some periods before the period of the Omicron variant. Moreover, there is no COVID-19 policy lag in Taiwan between daily new confirmed cases and each index. As of March 2022, the case fatality ratios of the elderly group in Japan, Hong Kong and South Korea are 4.69%, 4.72% and 1.48%, respectively, while the case fatality ratio of the elderly group in Taiwan is 25.01%. A government's COVID-19 vaccination distribution and prioritisation policies are pivotal for the elderly group to reduce the number of deaths. Immunising this specific group as best as possible should undoubtedly be a top priority.

## Introduction

Ever since the onset of the coronavirus disease 2019 (COVID-19) pandemic, many governments have implemented massive containment and other policies to preserve lives and enhance safety as well as introduced travel restrictions [1, 2] and numerous non-pharmaceutical interventions [3, 4] to bring down virus-related infections and deaths. Such interventions have also covered school closings, travel restrictions, bans on public gatherings and stay-at-home orders [5]. Many contemporary studies have thus examined government effectiveness targeting the COVID-19 pandemic at specific time points [6–8].

With COVID-19 already prevailing for almost 3 years, it is critical to assess government policies and their impact and effectiveness during this time. The present study assesses governments' long-term non-pharmaceutical interventions upon the COVID-19 pandemic in East Asia based on defined multiple pandemic waves. The study moves the literature towards a better understanding of when and which control measures are effective. We employ the Oxford COVID-19 Government Response Tracker (OxCGRT) datasets and narrow them down to East Asia for several reasons. First, the development of the COVID-19 pandemic in this region is very different from what has happened in Western countries. Second, this study offers a greater in-depth look and uncovers insightful information. Lastly, we investigate the proportion of new confirmed cases (new cases for short), new deaths and case fatality ratios of the elderly group.

## Data description

**Fig. 1.**
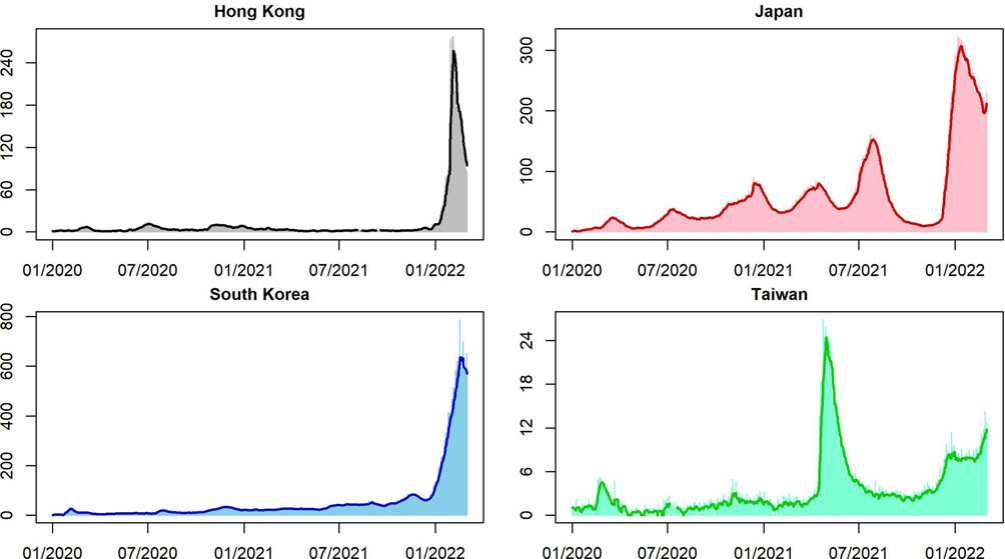
Square root of daily new confirmed cases (on the vertical line) and the square root of daily new confirmed cases smoothed by 7-day MA.

We collect the daily numbers of confirmed cases, numbers of deaths, containment and health index, stringency index, and government response index from Oxford COVID-19 Government Response Tracker [5] and further calculate daily confirmed cases and daily deaths by the 7-day moving average (MA) to allocate uncertain cases on the correct dates of the pandemic onset for some countries and region (Fig. 1). OxCGRT tracks individual policy measures across 20 indicators, producing four indices that aggregate daily information into a single number from 0 to 100. It measures how many relevant indicators a government has acted upon and to what degree. An index is an ordinal variable that is unable to provide whether a government's policy has been implemented effectively. This study scrutinises these indices associated with other information and uncovers insightful information. We describe the three indices as follows.

1. Containment and health index (CHI): the index integrates information on lockdown restrictions and related healthcare investments due to the pandemic. It relies on all ordinal containment/closure and health system policy indicators.
2. Stringency index (SI): the index measures the strictness of various lockdown policies that aim to reduce social activities and human contact. It covers all ordinal containment/closure policy indicators and an indicator on public information campaigns.
3. Government response index (GRI): the index records how government responses result in varying overall indicators in the OxCGRT database that become stronger or weaker throughout the outbreak. This index comes from all ordinal indicators. GRI provides a dynamic benchmarking measure for the speed of government responses globally to COVID-19.

**Fig. 2.**
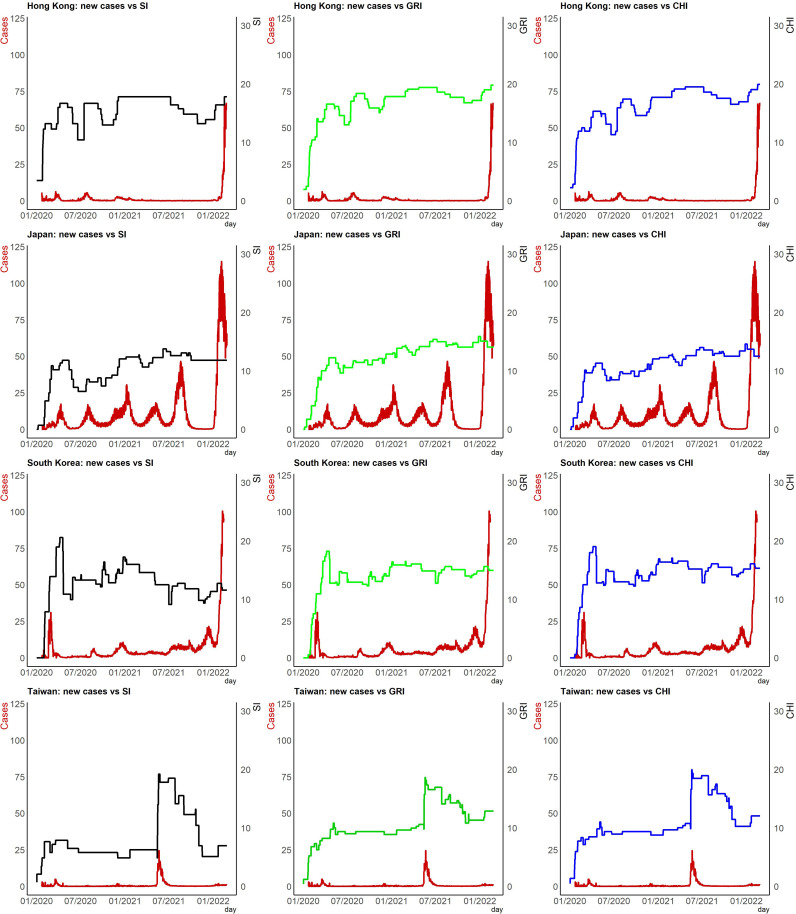
Time plots for rescaled daily confirmed cases associated with the indices.

Figure 2 illustrates the time plots of daily confirmed cases as of March 2022 associated with Japan, Hong Kong, South Korea and Taiwan indices. We rescale confirmed cases in the figure since the magnitudes are pretty large, as we cannot show their trend with the index in the same frame without doing so. The latest severe acute respiratory syndrome-coronavirus 2 (SARS-CoV-2) Omicron variant has spread worldwide since being first detected in South Africa on 9 November 2021. We clearly see that governments in East Asia have maintained or reintroduced strict measures designed in response to this variant's surge in 2022 to limit COVID-19's spread domestically. Population disparities among different countries and region are of course enormous. To conduct international comparisons, we collect total confirmed cases (deaths) of COVID-19 per million people and the proportion of the elderly group from ‘Our World in Data’ [9].

## Methodology

We examine the pairwise association between daily new cases (daily new deaths) and the indices. An appropriate measure is Kendall's coefficient of rank correlation or Spearman's rank correlation coefficient to evaluate the strength of linkage between two variables [10] when one variable is continuous and the other is ordinal. We use Spearman's rank correlation coefficient to see the strength of association between daily new cases (daily new deaths) and each index, because the index is a discrete variable having an order associated with its large number of levels. This helps us evaluate the effectiveness of governments' policies through the three indices.

We divide the whole sample period into several waves based on multiple domestic COVID-19 pandemic surges. As of 31 March 2022, Hong Kong has experienced five waves (see the appendix in [11] for the first four waves), while South Korea has dealt with four waves (see [12] for the periods of the first three waves). Japan has encountered its sixth wave ([13, 14] define the periods from the first wave to the fifth wave of the COVID-19 pandemic), while Taiwan has faced four waves based on the period of level 3.

**Fig. 3.**
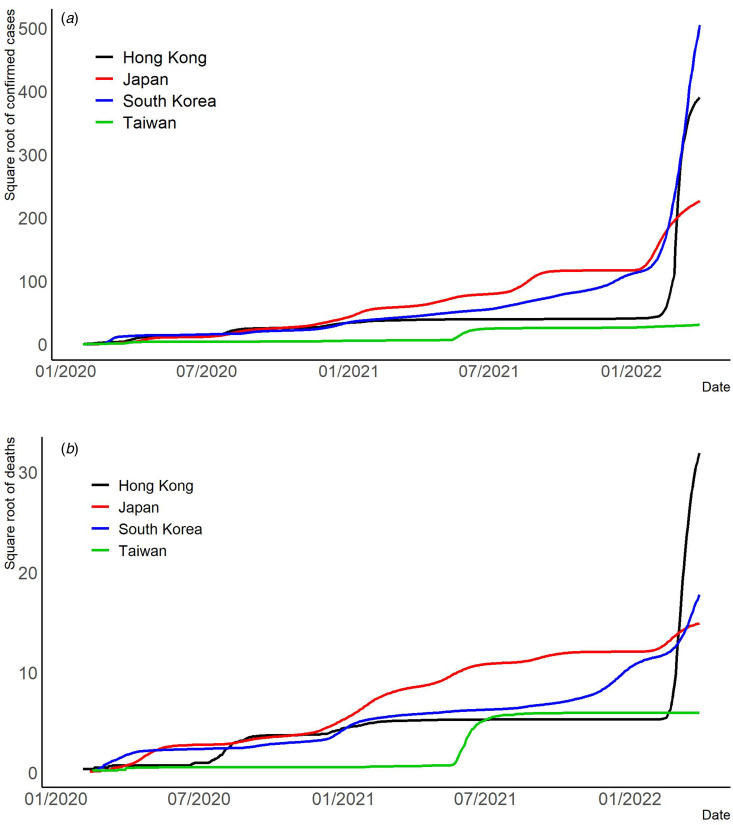
(a) Square root of the total confirmed cases of COVID-19 per million people. (b) Square root of the total deaths of COVID-19 per million people.

**Fig. 4.**
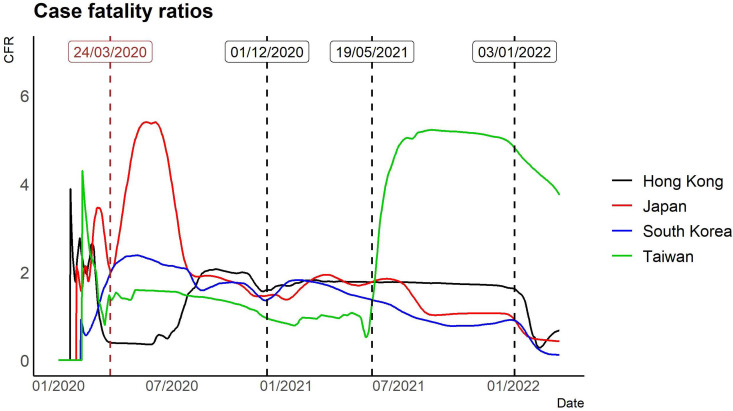
COVID-19 time-varying case fatality ratios based on the 7-day MA of confirmed cases and deaths.

We employ visualisation to help understand different aspects of the pandemic in East Asia. We also provide (1) time-varying rescaled (but the same scale for each country or region) daily new cases *vs.* each index (Fig. 2); (2) time-varying total confirmed cases of COVID-19 per million people (Fig. 3a); (3) time-varying total confirmed deaths of COVID-19 per million people (Fig. 3b) and (4) time-varying dynamic case fatality ratios (Fig. 4). We rescale confirmed cases in Figures 1–3 since their magnitudes are quite large.

**Fig. 5.**
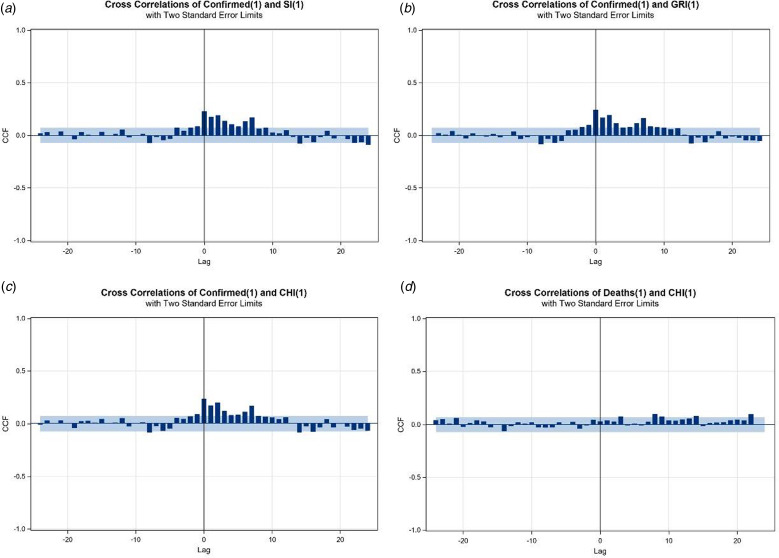
CCF plots based on pre-whitened series. (a)–(c) 7-day MA of confirmed cases *vs*. SI, GRI and CHI changes for Taiwan, respectively. (d) 7-day MA of deaths *vs*. CHI changes for Taiwan. The grey areas are the bands of two standard errors.

**Fig. 6.**
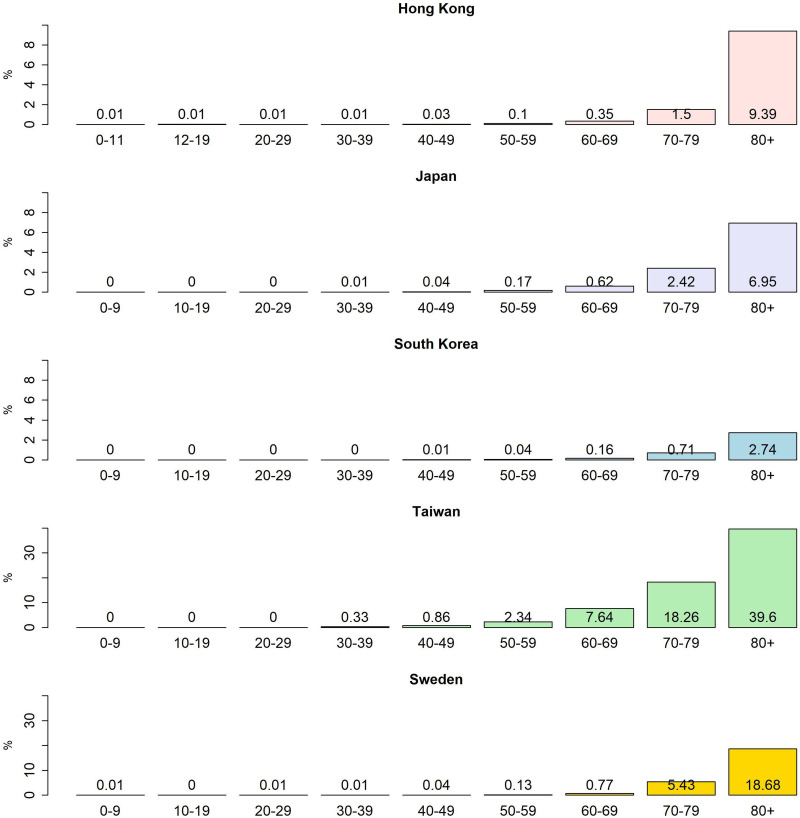
Case fatality ratios by age group in Japan, Hong Kong, South Korea, Taiwan and Sweden as of March 2022.

To explore the relationship between the series of SI, CHI or GRI and confirmed cases (deaths) of COVID-19, we apply the cross-correlation function (CCF) as an aid to identify the pattern of correlations. First, we conduct the first-differenced series of a considered policy index, named index changes, as a pre-whitening process. We then filter the 7-day MA of confirmed cases based on the model of the index changes. Following this strategy, the patterns of CCF between the daily new confirmed (daily new death) cases and index changes are easily identifiable (Fig. 5). The study calculates the age group-specific case fatality ratios by dividing the deaths from COVID-19 over a defined period by the number of confirmed cases during that time, especially focusing on the elderly group (Fig. 6).

## Results and discussion

To observe the co-movement of policy indices and rescaled daily confirmed cases, we provide the time series plots of these two variables together. We observe that the statuses of policy indices are associated with daily new cases in countries and region. We provide illustrative descriptions that appear in Figure 2.

*Hong Kong*: To stop the transmission of COVID-19 from overseas to Hong Kong, a mandatory quarantine was imposed for travellers from mainland China and South Korea in February 2020 and for travellers from Iran, Italy and other regions in March 2020 [15]. There were also restrictions on public gatherings implemented in April 2020. Therefore, it is not surprising that SI, GRI and CHI increased rapidly in the first 3 months of 2020. Since then, various hygiene measures for controlling the transmission of the disease have been introduced, and there was a slight upward trend in GRI and CHI after mid-2020. Early 2022 presented a sharp increase in confirmed cases, because of the transmission of the Omicron variant in Hong Kong. A few months before the spreading of Omicron, the three indices had increased slowly, probably indicating that there was a tightening of hygiene control measures in late-2021 after recording a few imported Omicron cases.

*Japan*: The number of confirmed cases surged, exceeding 200 across Japan as of 27 February 2020, excluding the over 700 confirmed cases from the Diamond Princess cruise ship. The Japan government adopted various measures to limit or prevent the outbreak in the first half of 2020, which included inaugurating a Joint Research Coronavirus Task Force to supervise its response to the pandemic and the suspension of all Japanese schooling from elementary to high schools from 27 February until early April 2020 [16]. On 7 April 2020, the Japan government proclaimed a 1-month state of emergency for Tokyo and several prefectures. The state of emergency was lifted for an increasing number of prefectures during May 2020, extending to the whole country by 25 May 2020 [17]. That is why we observe growth and a drop-down in Japan for SI, GRI and CHI in the first 6 months.

*South Korea*: After 18 February 2020, when the virus was initially confirmed in Daegu, the number of patients with COVID-19 increased rapidly. To respond to the rapid rise in the number of COVID-19 cases, the South Korea government puts the COVID-19 alert level at the highest (level 4) on 23 February 2020, enhanced social distancing measures and infection control measures in hospitals, imposed travel restrictions, cancelled social events and delayed the school start time [18]. We observe that SI, GRI and CHI shot up rapidly in the first 3 months of 2020. In February 2022, new confirmed cases surged to six-digit figures in South Korea.

*Taiwan*: COVID-19 infections cropped up only sporadically before May 2021. However, daily new confirmed case numbers soared into the triple digits on 15 May 2021. The Central Epidemic Command Center (CECC) raised level 3 COVID-19 alert for Taipei City and New Taipei City, strengthened national restrictions and measures, effective from 15 May, and further raised the epidemic warning to level 3 nationwide from 19 May 2021. Heightened measures and regulations were introduced across Taiwan to reduce community transmission [19, 20]. The government has implemented a mandatory wearing-mask policy, stringent restrictions on all kinds of gatherings, closure of entertainment venues and limiting restaurants to take-out service. CECC partially lifted certain restrictions for level 3 (starting on 13 July 2021 and ending on 26 July 2021). All these related policies reveal the rising and then plummeting SI, GRI and CHI during this period.

The Omicron variant was the predominant variant in Hong Kong, Japan and South Korea from late January 2022. We observe steep increase curves for daily new confirmed cases, while the Omicron outbreak in Taiwan is still in its early stages. The daily count of new cases surged as high as a few multiples of 10 000 outside this study's period (January 2020 to March 2022).

As the 7-day MA removes the fluctuation and derives a more stable trend, we provide Spearman's rank correlation coefficient to measure the association between the 7-day MA of new cases (new deaths) and each index (Tables 1–4). The results indicate the effectiveness of government policies through the specified range when the negative correlation is significant. Findings show significantly negative association measurements during different waves for Hong Kong, Japan, South Korea and Taiwan, but most effects are on confirmed cases with only a few situations on deaths. The exceptions appear on the fourth wave (1 July 2021 to 26 March 2022) in South Korea. Both confirmed and death cases in South Korea have significantly negative associations with the three indices (SI, GRI and CHI) during this period.

**Table 1.** Spearman's correlations between 7-day MA of new cases (new deaths) and policy indices for Hong Kong

Index	COVID-19 new	Whole period 28 January 2020–26 March 2022	First wave 28 January 2020–3 March 2020	Second wave 4 March 2020–21 June 2020	Third wave 22 June 2020–19 November 2020	Fourth wave 20 November 2020–6 April 2021	Fifth wave 31 December 2021–26 March 2022
SI	Case	−0.2278***	0.1363	−0.4827***	−0.3007***	−0.4446***	−0.3166**
	Death	0.0445	−0.1934	−0.0755	0.0277	−0.0738	0.2272*
GRI	Case	−0.1415***	0.0315	−0.5147***	−0.4264***	−0.1504	−0.2600*
	Death	0.0760*	−0.1394	−0.0748	−0.0162	−0.0406	0.2327*
CHI	Case	−0.1115**	0.1069	−0.5147***	−0.4280***	−0.1504	−0.2600*
	Death	0.0764*	−0.0811	−0.0748	−0.0293	−0.0406	0.2327*

*, **, and *** denote significance at the 5%, 1%, and 0.1% levels, respectively.

Governments did enforce stringent measures in the second and third quarters of 2020 after the COVID-19 virus broke out and quickly responded to the outbreak, encompassing restrictions on business activities, suspension of schooling, travel restrictions and social distancing rules (Fig. 3a). Interventions, such as enforced isolation, in Hong Kong did work truly well in the early pandemic to contain the virus over the second and third waves in terms of reducing confirmed cases (Table 1), as we find significantly negative associations between the three indices (SI, GRI and CHI) and daily confirmed cases. Since early 2022, Hong Kong has been experiencing huge and fatal virus surges via the highest total confirmed deaths of COVID-19 per million people in our study ([21] and Fig. 3b).

The number of confirmed cases increased substantially across Japan in late-February 2020. The Japan government adopted various hygiene measures to limit or prevent the outbreak in the first half of 2020. These could explain why we observe a significantly negative association between new confirmed cases and policy in the first wave in Table 2 for this country.

**Table 2.** Spearman's correlations between 7-day MA of new cases (new deaths) and policy indices for Japan

Index	COVID-19 new	Whole period 28 January 2020–26 March 2022	First wave 1 March 2020–30 June 2020	Second wave 1 July 2020–31 October 2020	Third wave 1 November 2020–28 February 2021	Fourth wave 1 March 2021–24 June 2021	Fifth wave 25 June 2021–30 September 2021	Sixth wave 6 January 2022–26 March 2022
SI	Case	−0.1314***	−0.3489***	−0.1700	−0.4970***	−0.4951***	−0.4807***	NA
	Death	−0.0210	0.2364**	−0.0079	−0.0696	−0.1294	−0.1689	NA
GRI	Case	0.0165	−0.4952***	−0.1700	−0.4558***	−0.4901***	0.2529*	0.6442***
	Death	−0.0304	0.0193	−0.0079	−0.1776	−0.1253	−0.4810***	0.2834*
CHI	Case	0.0206	−0.4040***	−0.1700	−0.4641***	−0.4901***	0.2529*	0.6442***
	Death	−0.0070	0.1892*	−0.0079	−0.1294	−0.1253	−0.4810***	0.2834*

*, **, and *** denote significance at the 5%, 1%, and 0.1% levels, respectively.

We observe evidence of the impact of non-pharmaceutical measures in controlling the spread of the virus in Japan during the whole study period except during the second and sixth waves (Table 2). The ‘Go To Travel’ campaign originally ran in the second half of 2020 to support the travel industry in Japan. We do not observe any significant association between new cases and the index in the second wave (Table 2). The subsidy programme was suspended on 28 December 2020, complicating government efforts to address the economic fallout from the pandemic and extending the State of Emergency during the third wave. This may explain the negative association between new cases and SI, GRI and CHI during the third and fourth waves. Daily new cases in Japan soared following the start of the Olympics in the last week of July and in early August of 2021. Consequently, the policy indices of GRI and CHI fail to show effective prevention of confirmed cases in the fifth wave. Because of a state of emergency declaration and a spectator-less Tokyo 2020 Olympic Games, the number of confirmed cases in Tokyo dropped after the fifth wave. The negative association between new cases and SI also confirms this fact. Currently, in the sixth wave Japan has seen a rapid increase in new confirmed cases by more than three times over the previous wave, fuelled by the surge of Omicron. None of the indices negatively affect new confirmed cases in the sixth wave.

The economic reactivation and public health control of the COVID-19 pandemic have been asymmetric. When social activities were reactivated to boost the economy, the second and third waves in South Korea started, but there were differences in the public health response [12]. Our results of the second and third waves in Table 3 confirm a negative association between new cases and each index in the third wave, while a positive association exists between new cases and each index in the second wave. The results reveal South Korea's trending down of new death cases in the fourth wave and the entire period (Table 3). South Korea presents two seemingly contradictory pandemic indicators. It recorded over 100 000 new confirmed cases every day from 17 February 2022 to March 2022, as seen from the trend in Figure 3a. At the same time, the country has one of the lowest virus death rates globally (https://covid19.who.int/table).

**Table 3.** Spearman's correlations between 7-day MA of new cases (new deaths) and policy indices for South Korea

Index	COVID-19 new	Whole period 28 January 2020–26 March 2022	First wave 1 February 2020–31 March 2020	Second wave 13 August 2020–18 September 2020	Third wave 4 November 2020–31 January 2021	Fourth wave 1 July 2021–26 March 2022
SI	Case	−0.2290***	−0.0173	0.7060***	−0.4876***	−0.1461*
	Death	−0.1734***	0.1114	−0.0434	−0.0949	−0.3055***
GRI	Case	−0.0732*	−0.0717	0.7060***	−0.5573***	−0.1364*
	Death	−0.1018**	0.0883	−0.0434	−0.1953	−0.2909***
CHI	Case	−0.0836*	−0.0843	0.7060***	−0.5573***	−0.1364*
	Death	−0.1060**	0.0827	−0.0434	−0.1953	−0.2909***

*, **, and *** denote significance at the 5%, 1%, and 0.1% levels, respectively.

During the first wave, Taiwan had nearly unblemished success at keeping the COVID-19 pandemic at bay with the world's longest track of case-free days. Due to the outbreak of infections derived from tea houses in the red-light district of Wanhua in Taipei on 23 April 2021, Taiwan was in the grip of its first major COVID-19 surge. The CECC announced a nationwide level 3 epidemic alert on 19 May 2021, but later eased some restrictions for level 3 from 13 July to 26 July 2021. The intervention results during the second wave seem to have been not so effective, showing insignificant associations between cases (deaths) and each index. The intervention during the third wave by Taiwan was relatively significant compared to the previous waves (Table 4).

**Table 4.** Spearman's correlations between 7-day MA of new cases (new deaths) and policy indices for Taiwan

Index	COVID-19 new	Whole period 28 January 2020–22 March 2022	First wave 28 January 2020–22 April 2021	Second wave 23 April 2021–26 July 2021	Third wave 27 July 2021–2 January 2022	Fourth wave 3 January 2022–22 March 2022
SI	Case	−0.1989***	−0.0618	−0.1162	−0.2540**	−0.2131
	Death	−0.0328	0.0000	−0.0517	−0.1111	0.0000
GRI	Case	−0.0755*	0.0281	−0.0355	−0.1607*	−0.2392*
	Death	−0.0285	0.0259	0.1413	−0.1118	−0.0248
CHI	Case	−0.0814*	0.0264	−0.1163	−0.1828*	−0.2392*
	Death	−0.0342	0.0259	−0.0524	−0.1118	−0.0248

*, **, and *** denote significance at the 5%, 1%, and 0.1% levels, respectively.

A lead–lag effect, especially in economics, describes when one (leading) variable cross-correlates with the values of another (lagging) variable at later times. This study aims to quantify the time-lag effect reflected in new confirmed cases from authorities' response to the COVID-19 pandemic using SI (GRI or CHI) as an indicator of policy effectiveness. From the healthcare perspective, there are COVID-19-related studies that do investigate the time lag between the mobility response and government policy [22] and the lead–lag relationship between physical and mental health [23]. Our findings show no policy lag in Taiwan (Fig. 5). At the same time, there is no significant relationship between new confirmed cases and each index for Hong Kong, Japan and South Korea.

In May 2020 14% (79 out of 557) of COVID-19-related deaths in Japan took place in long-term care facilities [24]. Long-term care facilities with elderly residents are especially high-risk facilities in the event of COVID-19 viral transmissions. This may explain why the case fatality ratio of Japan is relatively higher than in other East Asia countries during the first wave (Fig. 4). For comparison, we also include the case fatality ratio of the elderly group for Sweden in Table 5. By the beginning of December 2020, more than 7000 people had died of COVID-19 in Sweden. Of these, almost 90% were 70 years or older, half were living in a long-term residential care facility, and just under 30% received home help services [25].

**Table 5.** Information on the elderly group and test (per 1000) for all ages

	Confirmed cases	Deaths	Case fatality ratio	Test (per 1k)	Proportion of elderly
	Age ≥70 (%)	Age ≥70 (%)	Age ≥70 (%)	All ages	Age ≥65 (%)
Hong Kong	11.63	87.51	4.72	5954.47	19.60
Japan	8.18	86.53	4.69	328.57	29.79
South Korea	6.93	81.21	1.48	1683.60	16.65
Taiwan	13.27	61.62	25.01	292.77	15.96
Sweden	5.66	88.75	11.50	1788.10	20.10

The case fatality ratios by age group in Japan, Hong Kong, South Korea and Taiwan as of March 2022 appear in Figure 6.^1^ This confirms an earlier report [25] that case fatality ratios exhibit age group-specific disparity and a high percentage of deaths in elderly people. It shows that the case fatality ratios of the elderly group (age ≥70) in Japan, Hong Kong and South Korea are 4.69%, 4.72% and 1.48%, respectively, while the case fatality ratio of the elderly group (age ≥70) in Taiwan is 25.01% (Table 5). The proportion of deaths in the elderly group (age ≥70) of Japan as of March 2022 is 86.53% of confirmed deaths, in which the first wave may dominate. The reason for elderly deaths could be that some countries performed mass testing or initiated substantial prevention measures, which helped avoid a large number of potential deaths; it could also be that insufficient vaccinations led to them being unable to prevent these issues or families abandoning their elderly amid this pandemic [26].

We also provide total tests per thousand (total tests/population × 1000) as of March 2022 in the fifth column of Table 5. Hong Kong and South Korea have undergone mass testing compared to Japan and Taiwan. At the start of the pandemic in 2020, South Korea was hit by some of the worst early COVID-19 outbreaks, but it appeared to have brought them under control through mass testing and aggressive contact tracing. However, the South Korea government announced on 7 February 2022 that it was abandoning the test-and-trace system in the face of a surge of Omicron cases that threatened to overwhelm the country's health system [27]. Authorities of South Korea prioritise tests for people aged 60 or older.

We add the proportion of elderly in East Asia and Sweden in the last column of Table 5. Unfortunately, we only can provide the proportion of people aged over 65 in 2021 instead of the elderly group aged over 70. We observe that the proportion of elderly people in 2021 ranges from around 15% to 30% and also note that the proportion of the elderly in Japan is the highest in this study. There is clearly no obvious association between this proportion and the case fatality ratio for the elderly group.

**Fig. 7.**
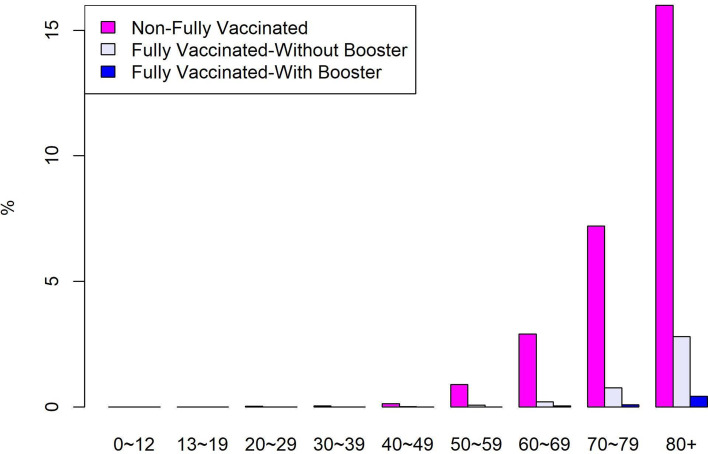
Proportion of COVID-19 cases in Singapore who died, by age and vaccination status.

It is impossible to examine how the Hong Kong and Taiwan governments are responding to COVID-19 without considering their past experience with the SARS epidemic in 2003. Hong Kong and Taiwan both faced severe consequences from the SARS outbreak [28, 29]. Both governments have effectively provided non-pharmaceutical interventions in terms of the negative association between COVID-19 confirmed cases and the indices. Table 1 reveals such negative associations between COVID-19 confirmed cases and the indices of Hong Kong during the second, third and fifth waves. However, the proportion of deaths in the elderly group (age ≥70) of Hong Kong is 87.51% of confirmed deaths, which is similar to Sweden's elderly group at 88.75% (Table 5). As of 25 March 2022, the proportion of people aged over 80 in Hong Kong who have received two vaccine doses or more is 53.19% [30]. Around one-half of people aged 80 and over in Hong Kong have been given two doses or more of a vaccine before the Omicron surge, compared to over 90% in Singapore. A recent report (accessed 27 March 2022) by the Ministry of Health, Singapore shows that 16% of confirmed death in those aged 80 and over in Singapore were non-fully vaccinated, while 2.8% and 0.42% were fully vaccinated-without booster and fully vaccinated-with booster, respectively (Fig. 7).

There is a steep case fatality ratio for Taiwan after May 2021. The case fatality ratio of March 2022 in the elderly group (age ≥70) is 25.01%, which is the highest case fatality ratio for the elderly group in our study. Taiwan began Oxford-AstraZeneca COVID-19 vaccinations on 22 March 2021, while the Moderna COVID-19 vaccine rollout started on 9 June 2021. Its CECC announced a COVID-19 vaccine distribution strategy on 20 June 2021. Table 6 presents the 10 approved priority groups.

**Table 6.** COVID-19 vaccination rollout in Taiwan announced by the CECC on 20 June 2021

Group	Vaccination target	Estimated number of people^a^	Cumulative number of people^a^
1	Healthcare workers	50.6	50.6
2	Central and local government epidemic prevention personnel	13.9	64.5
3	Frontline workers with a high risk of COVID-19; e.g. domestic aviation industry flight crew, etc.	6.1	70.6
4	Those who need to travel abroad	0.2	70.8
5	Law enforcement officers and firefighters	46.7	117.5
6	Elderly people aged 75 and over and pregnant women	168	285.5
7	National security personnel	86	371.5
8	Those aged 65–74	198.5	570
9	Adults aged 19–65 with life-threatening conditions, rare diseases or a history of serious illness	387.5	957.5
10	Adults between the ages of 50 and 64	530	1487.5

a Unit is 10 000 people.

The elderly people aged 75 and over and pregnant women rank in the sixth priority group, while those aged 65–74 ranked as the eighth priority, and adults aged 50–64 with the largest number rank at tenth priority. This may explain the highest case fatality ratio of March 2022 in Taiwan in the elderly group, as they are insufficiently vaccinated. Based on the death rate of Hong Kong's largely unvaccinated elderly group [21] and the lower priority order for Taiwan's elderly group after vaccines rolled out, we draw a conclusion that a fully vaccinated person with a booster is presently the best strategy for reducing deaths from COVID-19 in the elderly group.

## Limitations

This study has two limitations. First, the information provided in this study relates to current evidence but may be modified as more information becomes available. Second, the number of confirmed cases may be lower than the true number of infections due to limited testing.

## Conclusion

Our results show that non-pharmaceutical interventions have significant effects on reducing COVID-19 infections for some periods before the rise of the Omicron variant, offering evidence of a significantly negative association between confirmed cases and the study's indices. The three indices do not include an evaluation of vaccination, and CHI only covers investments in vaccines. Our conclusion agrees with some recent studies [8, 31] in which advances in the prevention and effective management of COVID-19 will require basic and clinical investigation and public health and clinical interventions. A government's COVID-19 vaccination distribution and prioritisation policies are pivotal for the elderly group. The study offers a particularly profound lesson, whereby if the vaccination rate for the elderly is low, then their death rate tends to be high. Immunising the elderly group as best as possible and managing their ‘long COVID’ after infection [32] have undoubtedly become top priorities.

## Data Availability

The dataset underlying the results described in this paper can be found in the ‘Data description’ section. Details of all data sources can be found in the Supplementary material.

## Footnotes

1 All age group data sources are from government official websites as of 25–27 March 2022.

## Supplementary material

For supplementary material accompanying this paper visit http://doi.org/10.1017/S0950268822001388. click here to view supplementary material
