# Supplementary material for: Assessing government policies' impact on the COVID-19 pandemic and elderly deaths in East Asia
Source: Epidemiol Infect. 2022 Aug 22;150:e161. doi: 10.1017/S0950268822001388 (PMC9472036; doi:10.1017/S0950268822001388)
Supplement: Supplementary file 1 [file S0950268822001388sup001.pdf]

Data Availability Statement

Tables 1-4 and Figure 4:

In Tables 1 to 4 and Figure 4 the data source of the 7-day MA of new cases (7-day MA of new deaths) and policy indices is the Oxford Covid-19 Government Response Tracker (OxCGRT) by the link: https://github.com/OxCGRT/covid-policy-tracker/raw/master/data/OxCGRT_nat_latest.csv (accessed 30 March 2022).

The file has been updated continuously. The data used in this study are as of March 2022 and can be found from the historical data in the link.

Table 5 & Figure 6:

In Table 5 and Figure 6 we obtain age group data from the following websites.

Hong Kong:

https://www.coronavirus.gov.hk/eng/5th-wave-statistics.html, (accessed 26 March 2022).

https://www.coronavirus.gov.hk/pdf/5th_wave_statistics/5th_wave_statistics_20220325.pdf, statistics of the 5th wave of COVID-19, Centre for Health Protection of the Department of Health; the age distribution of confirmed cases/deaths is as of 25 March 2022.

Japan:

https://covid19.mhlw.go.jp/extensions/public/en/index.html, Ministry of Health, Labor and Welfare (accessed 22 March 2022).

South Korea:

http://ncov.mohw.go.kr/cn/bdBoardList.do, Central Epidemic Prevention Countermeasure Headquarters (accessed 22 March 2022).

Taiwan:

https://sites.google.com/cdc.gov.tw/2019ncov/taiwan (accessed 26 March 2022).

Sweden:

https://www.statista.com/statistics/1107913/number-of-coronavirus-deaths-in-sweden-by-age-groups/, Number of coronavirus (COVID-19) deaths in Sweden, by age groups (accessed 29 March 2022).

Figure 1 & Figure 2:

In Figures 1 and 2 the data source of daily new confirmed cases and policy indices is the Oxford Covid-19 Government Response Tracker (OxCGRT) by the link:

https://github.com/OxCGRT/covid-policy-tracker/raw/master/data/OxCGRT_nat_latest.csv

(accessed 30 March 2022). The file has been updated continuously. The data used in this study are as of March 2022 and can be found from the historical data in the link.

Figure 3:

In Figure 3 the data source of total confirmed cases or total deaths of COVID-19 per million people for all countries and region is from the website of Our World in Data (https://github.com/owid/covid-19-data/raw/master/public/data/owid-covid-data.csv). The data file has been updated daily. In our study the data used are as of March 2022 and can be found from the historical data in the link.

Figure 5:

For Figure 5 the data source of daily new confirmed cases, daily new deaths, and policy index (SI, GRI, and CHI) changes is from the Oxford Covid-19 Government Response Tracker (OxCGRT) by the link: https://github.com/OxCGRT/covid-policy-tracker/raw/master/data/OxCGRT_nat_latest.csv (accessed 30 March 2022). The file has been updated continuously. The data used in this study are as of March 2022 and can be found from the historical data in the link.

Figure 7:

In Figure 7 the data source of "Proportion (%) of cases who died, by age and vaccination status is the link: https://www.moh.gov.sg/covid-19/statistics/, accessed on 27 March 2022. The information in this figure is based on the period from 1 May 2021 to 28 February 2022.

Table 6:

We obtain the COVID-19 vaccine distribution strategy for Taiwan announced by CECC from the website: https://www.taiwannews.com.tw/en/news/4214853
